# How admixed captive breeding populations could be rescued using local ancestry information

**DOI:** 10.1111/mec.17349

**Published:** 2024-04-18

**Authors:** Daniel J. Lawson, Jo Howard‐McCombe, Mark Beaumont, Helen Senn

**Affiliations:** ^1^ Institute of Statistical Sciences, School of Mathematics University of Bristol Bristol UK; ^2^ RZSS WildGenes Laboratory, Conservation Department Royal Zoological Society of Scotland Edinburgh UK; ^3^ School of Biological Sciences University of Bristol Bristol UK

**Keywords:** admixture, captive populations, conservation genetics, hybridization

## Abstract

This paper asks the question: can genomic information be used to recover a species that is already on the pathway to extinction due to genetic swamping from a related and more numerous population? We show that a breeding strategy in a captive breeding program can use whole genome sequencing to identify and remove segments of DNA introgressed through hybridisation. The proposed policy uses a generalized measure of kinship or heterozygosity accounting for local ancestry, that is, whether a specific genetic location was inherited from the target of conservation. We then show that optimizing these measures would minimize undesired ancestry while also controlling kinship and/or heterozygosity, in a simulated breeding population. The process is applied to real data representing the hybridized Scottish wildcat breeding population, with the result that it should be possible to breed out domestic cat ancestry. The ability to reverse introgression is a powerful tool brought about through the combination of sequencing with computational advances in ancestry estimation. Since it works best when applied early in the process, important decisions need to be made about which genetically distinct populations should benefit from it and which should be left to reform into a single population.

## 1 INTRODUCTION

Hybridization between threatened species and more abundant relatives can lead to species extinction and is exacerbated by human activity, so it is natural to ask if this can be slowed or reversed. Reversing hybridization is conceptually difficult and hotly debated; Powell (2023) argued that we cannot, and should not, ‘unscramble the egg’ and control hybridization in a conservation context, an issue which is hotly debated (Chan et al., 2019; Hirashiki et al., 2021; Jackiw et al., 2015; vonHoldt et al., 2018) on both practical and ethical grounds. Species that can hybridize may be considered as populations of a higher‐order species‐complex, and viewed this way, hybridization simply mixes historical populations and so may beneficially increase genetic diversity, without necessarily negative consequences for populations in the *future*. However, reducing anthropogenic hybridization (caused by domestic species or human‐mediated introduction of wild species) is easier to advocate (Ottenburghs, 2021). Hybridization of a threatened species with one domestic or human‐associated can cause extinction by genetic swamping (replacement of lineages by the more abundant species) or demographic swamping (low fitness of hybrids due to outbreeding depression) (Todesco et al., 2016). This is especially important if the threatened species has an ecologically functional ecosystem role their more common domestic counterparts do not perform.

Deintrogression, that is the process of removing unwanted genetic material from a threatened species by breeding, has been previously proposed using genomic information (Amador et al., 2014), but existing strategies lead to a heavy diversity cost which has dissuaded action in practice. We will show that this constraint can be lifted by the estimation of ‘local ancestry’, that is identifying the ancestry ‘localised’ to a particular section of the genome. This is the first practical mechanism for reversing hybridization in a breeding program, though we emphasize that it may not be appropriate to do so in any particular case.

Measuring local ancestry provides a revolutionary ability to breed‐out past introgressed genomic segments. We propose an ancestry‐aware genomic analogue of the traditional kinship coefficient, which is the probability that both copies of a randomly chosen allele (i.e. variant of a genomic feature that varies between individuals) are identical by descent (IBD), here from a recent founder of the breeding population. This can be directly used in place of the pedigree in breeding selection (Speed & Balding, 2015). The price is that instead of tracking a traditional pedigree (Pemberton, 2004) the approach requires genomic information. Previous discussion in conservation typically compares pedigrees to low‐density markers (Hauser et al., 2022; Norman et al., 2018; Wang, 2016), but this approach requires high‐density markers in strong linkage disequilibrium (LD), most likely whole genome sequencing data.

Ancestry inference using software such as STRUCTURE (Pritchard et al., 2000) and ADMIXTURE (Alexander et al., 2009) is common in conservation (Wright et al., 2021) using low density (i.e. mostly independent) genetic markers such as single nucleotide polymorphism (SNP) arrays or microsatellites (Ivy & Lacy, 2012), and targeted sequencing (Hogg et al., 2018) such as ddRAD (Ivy et al., 2016). However, high‐density markers (i.e. hundreds of thousands or more) are correlated due to LD which allows the identification of which variants were jointly inherited (Browning & Browning, 2007). Consequently, shared haplotypes—that is contiguous DNA shared by descent between multiple individuals—can be identified from the same recent ancestor. Local ancestry specific to segments of each haplotype (Lawson et al., 2012; Price et al., 2009) can then be inferred, for which we use the software MOSAIC (Salter‐Townshend & Myers, 2018), allowing detailed inference of population structure and identification of regions with anomalous ancestry indicating selection, for example in honeybees (Nelson et al., 2017) and wildcats (Howard‐McCombe et al., 2023; Jamieson et al., 2023). These tools are important for understanding population structure (Lawson & Falush, 2012) but so far have had little impact in conservation.

Captive breeding programs typically operate with smaller populations than would be ideal to maintain long‐term population viability (Akcakaya & Sjogren‐Gulve, 2000; Chaudhary & Oli, 2020). The retention of genetic diversity is therefore the focus. Ideally conservation measures would target an effective population size (*N*_*e*_) of at least 500 (Frankham et al., 2014), a threshold used by the Convention on Biological Diversity (CBD) (Hoban et al., 2020) to describe a healthy population. The fixation of rare and potentially maladaptive alleles can lead to disproportionate heritable disease burden in small populations, for example inbreeding depression in Italian wildcats (Lioy et al., 2022). For this reason, breeding management—as exemplified by the popular PMx software (Ivy & Lacy, 2012) for decision support, as well as Vortex (Lacy, 2000) for population viability analysis—emphasize minimizing kinship. Kinship is the proportion of the genome that is identical due to recent shared relatives between parents of an individual (Ballou et al., 1995). Kinship is not the only way to measure inbreeding, with alternatives including using (sparse) molecular markers to measure allele sharing (Ivy et al., 2016) or heterozygosity (Wright et al., 2021), but these incompletely characterize variation.

Endangered species that experience anthropogenic hybridisation with more numerous species, typically domestic or human‐associated, are under threat of extinction via genetic swamping (Rhymer & Simberloff, 1996; Todesco et al., 2016), that is the replacement of their genome by that of a more numerous related species or population. Partridges (*Alectoris graeca* and *A. rufa*) and wolves (*Canis lupus*) (Randi, 2008) experience this problem, and it is a potential threat to captive breeding programs including African penguins (*Spheniscus demersus*) (Modesto et al., 2018), crocodiles (*Crocodylus siamensis* and *C. porosus*) (Lapbenjakul et al., 2017), Bison (*Bison bison*) (Stroupe et al., 2022) and in our case, the Scottish wildcat *Felis silvestris*. Such species pose a particular challenge for conservation because the breeding program may not have been in place before hybridization began. This holds true for the Scottish wildcat, where captive‐breeding cats possess an average of 18% admixture from domestic cats, and an even higher proportion is observed in the remaining wild population (Senn et al., 2019). There are no unadmixed individuals, but we have recently shown (Howard‐McCombe et al., 2023) that a near‐complete genome exists across the population. We will show that if it is deemed desirable to do so, hybridization of this magnitude and timescale can be recovered to close to pre‐hybridization levels using local ancestry inferred for dense markers.

The challenge for conservation in such cases is that there is currently no tool to remove introgression. As shown by Amador et al. (2013), directly minimizing hybridization is possible but greatly accelerates diversity loss, whilst controlling kinship alone will not remove the introgressed genome. Trying to maximize genetic diversity using molecular estimators could paradoxically make things worse due to the introgressing species being genetically distinct from the target species, so that selection for diversity increases the frequency of introgressed alleles. Few measures suitable for breeding choices while accounting for local ancestry are available. Much prior work focuses on the estimation of heterozygosity of local ancestry itself, for example (Fitzpatrick, 2012; Maples et al., 2013). Heterozygosity of F2 hybrids has been used to compute hybrid incompatibility (Thompson et al., 2022), and runs of homozygosity have been decomposed by local ancestry (Szpiech et al., 2019). Ancestry‐specific *F*_*ST*_ has been linked to kinship (Ochoa & Storey, 2021) for the purpose of estimating population structure. In contrast to previous work (Amador et al., 2014), we will introduce a local ancestry ‘mask’ for computing ancestry‐specific heterozygosity or kinship in admixed individuals.

We focus discussion around the Scottish wildcat, the north‐westernmost population of the European wildcat *Felis silvestris* which is distributed in fragmented populations across Europe. It is related to the domestic cat that evolved from *Felis lybica*, which is distributed across north Africa and the middle east. Domestic and European wildcat species are genetically quite distinct, their progenitor populations having diverged around 1M years ago (Li, Davis, et al., 2016), with *F*_*ST*_ = 0.46 (Howard‐McCombe et al., 2023) and therefore the two species are quite separable. In Britain, the species remained reproductively isolated until the mid‐1900s, even though both species were present since the introduction of the domestic cat 2800–2200 years BP, which implies considerable differences in lifestyle and/or behaviour. Wildcat populations in Britain declined as a result of hunting for sport and fur, persecution as vermin and habitat loss (Langley & Yalden, 1977), becoming extinct from England and Wales by late 1800's and becoming almost extinct in Scotland by around 1915. Populations recovered after WW1 as the reduction in gamekeeping reduced persecution and encouraged re‐afforestation, with a further decline again from the 1960's–1970's onwards as threats re‐established. Hybridisation with domestic cats became an active concern from the 1980's onwards (French et al., 1988). A UK studbook, which is both a historical and living dataset of all wildcats held in captivity, has been in existence since the late 1990s. In 2015 following the launch of the Scottish Wildcat Conservation Action Plan (Scottish Natural Heritage, 2013), the studbook was managed by the Royal Zoological Society of Scotland (RZSS). Since 2016, the population has been managed for hybridisation by excluding individuals with more than 25% domestic ancestry based on 35 highly differentiated genomic markers that together quantify genome‐wide admixture. The studbook is managed using standard population management methodologies implemented in PMX. Joint analysis of genetic data and studbook records indicates there are approximately 26 founders and that there are descendants from animals taken into captivity up to 45 years ago, however there are significant gaps in earlier record keeping. First releases from the breeding programme began in June 2023 as part of the Saving Wildcats Project.

The objectives for this paper are: firstly, to develop genomic measures based on local ancestry that can be used for selective breeding that can perform deintrogression in principle. Secondly, to be able to recommend at least one procedure that should work in practice. Thirdly, to be able to estimate the timescale that deintrogression might take, based on the history of introgression that has occurred. And finally, to set out a pathway for this to be implementable. We will test the proposed procedure by simulating breeding programs forward in time, and measuring both traditional performance measures (average ancestry, kinship and heterozygosity) as well as the ancestry‐specific versions. This will be done for a range of simulation parameters, as well for a population constructed from Scottish Wildcat data.

**FIGURE 1:**
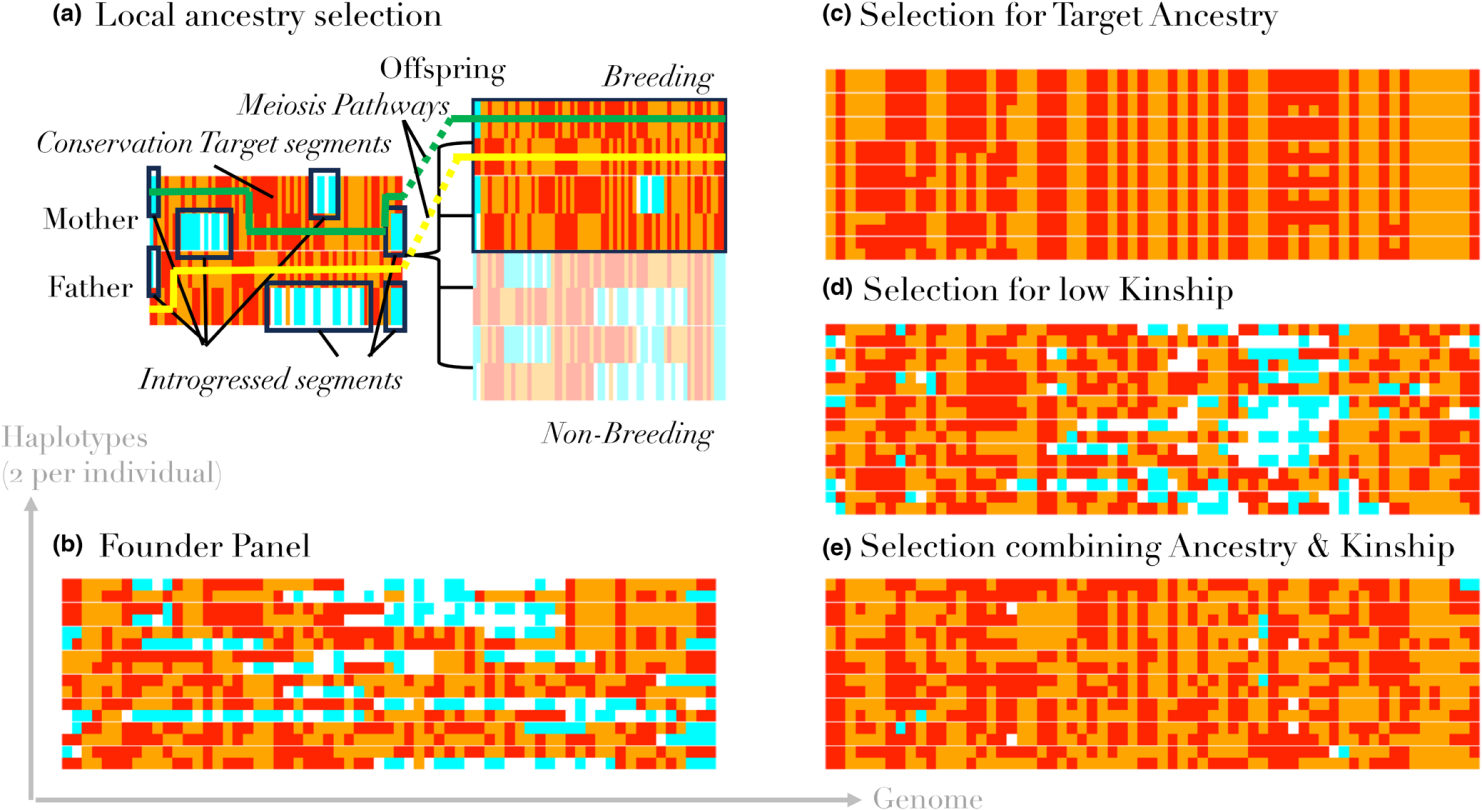
Concepts in this paper. (a) The process of meiosis takes the two haplotypes (rows) from each parent and makes a single random haplotype; the yellow and green lines are one possible realization of SNPs (columns). The conservation target ancestry (orange, red with mutation) can be separated from introgressed ancestry (white, cyan with mutation). This allows selection of the most suitable offspring for the next generation, who each receive different random haplotypes. (b) Initially we start with a relatively diverse and introgressed panel, with each individual consisting two rows (haplotypes). (c) If we select against introgression, we lose diversity in the genome. (d) If we select to minimize kinship, we fail to remove introgression. (e) If we select for diversity with knowledge of local ancestry as in (a), we can balance both pressures.

Figure 1 illustrates how the approach works. We observe that recent developments in local ancestry estimation could be used for a breeding program. Methods for estimating ancestry from whole‐genome data are reliable in well‐diverged species of conservation interest (discussed in Section 3.1), so we can assume that the ancestry status of haplotypes are known (demonstrated for wildcats in Figure S1). We therefore use simulation to examine how this can be used in a breeding program. We examine different choices of selection measure for the breeding program, and consider the genetic diversity consequences for the species. Existing breeding‐decision rules for kinship or heterozygosity can be extended to use this local ancestry information by ‘masking’ out ancestry from the undesired species, which reduce to familiar measures in the absence of introgression. Our simulation suggests that correct management using our proposed local ancestry measures can turn around the introgression status of a population in many circumstances.

## 2 METHODS AND RESULTS

### 2.1 A simulated breeding program

This section contains a complete definition of the simulation and inference procedure in words. Formal mathematical descriptions are referenced in the Methods Appendix (Section 4). The experimental design considers a hybridized captive population with genetic components from two original sources, the ‘target’ population for conservation and the ‘introgressing’ population. Section 2.2 considers default parameters appropriate for the Wildcat breeding program, before undertaking a sensitivity analysis to parameters that may be seen in other populations (Section 2.3). Wildcat genomic data is examined in Section 2.4.

We initialise a founder captive breeding population (Section 4.9) of *N* = 150 diploid individuals in which two generations of admixture occurred 10 generations before the captive population was formed. The target and introgressing populations are separated by an *F*_*ST*_ = 0.4, that is if a SNP has overall frequency $f_{l}$, the variance between populations is $F_{\mathrm{ST}}f_{l}\left(1-f_{l}\right)$, under the Balding‐Nichols model (Balding & Nichols, 1995) of genetic drift. Every generation before the breeding program starts, mate choices are made randomly under the Wright‐Fisher process with the genetic meiosis model described below. There are two generations in the establishment of the captive population, the first being a bottleneck due to sampling wild individuals in which only a fraction (25% here) of individuals are sampled for the breeding program. In the second generation, the least introgressed proportion (25%) of captive individuals are retained.

**FIGURE 2:**
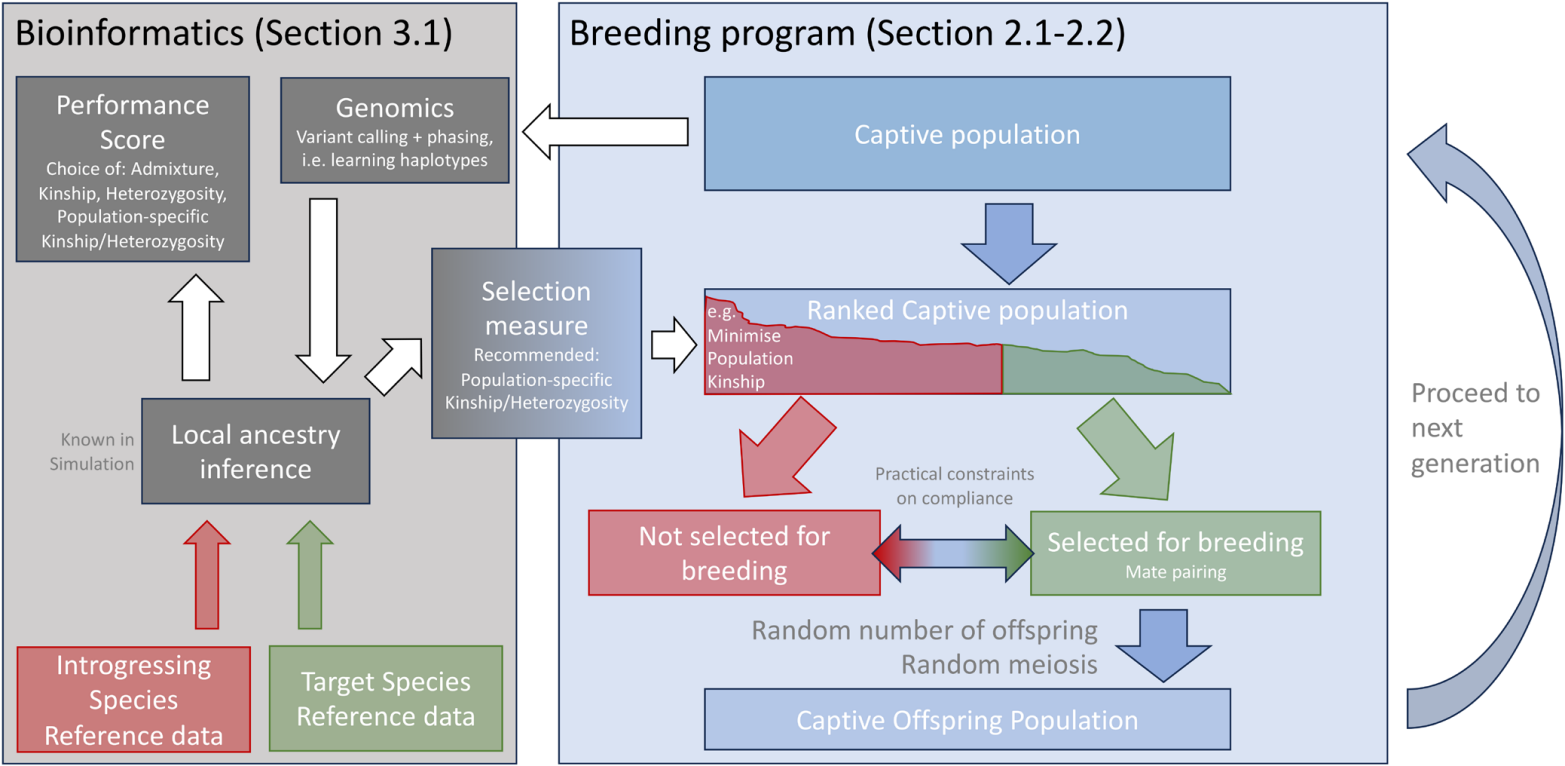
Conceptual diagram of the breeding program. Left: bioinformatics needed to obtain the local ancestry information required for breeding selection in practice (see Section 3.1). Right: the simulated breeding program, as explained in Section 2.1, with selection measures explored in Section 2.2. Dark text relates to details in the simulation that would be determined externally in a real breeding program.

We simulate the captive population forwards in time as illustrated in Figure 2 (right):

1. **Local ancestry information** for each individual in the program is obtained. In a simulation this is given; Section 3.1 expands the bioinformatics required for real data (Figure 2 left).

2. **Individuals are ranked** based on the ‘ranked mean’ selection procedure (Section 4.10) which iteratively ranks individuals from ‘best’ to ‘worst’ in terms of their expected contribution to the next generation. The selection measure is an average over the higher‐ranked individuals who are more likely to have offspring, and is also averaged over the *L* observed loci on the genome (*L* = 500 by default), as described in Section 2.2. We will assume that this process is not perfect such that breeders can only comply with a proportion of recommendations (Section 4.10.2). Ranked mean has been shown to be appropriate for kinship (when it is called the ‘ranked MK’ procedure (Ivy & Lacy, 2012)) if breeding occurs in discrete generations.

3. **Offspring are generated** for each pair:

(a) **The number of offspring is random** (Poisson distributed with mean 4.2), from the best pairs downwards, until the program reaches capacity in the next generation (here *N* = 150).

(b) **Meiosis is simulated** with each offspring individual formed by inheriting one randomized gamete from each parent (Figure 1). A gamete is formed by inheriting segments from one of the two copies of a parents' genome, after which it ‘crosses over’ to the other copy. Segment have length ~exp(1), measured in the recombination unit Morgans, meaning one recombination per generation is expected. By default we use a genome of length 5 Morgans.

### 2.2 Breeding selection

The critical component of a breeding program is mate selection. We first consider three traditional breeding measures which contain information that can be acquired without local ancestry. As a baseline, we consider choosing individuals randomly (*R*). This is compared to breeding selection measures which optimize an *expected score* in the offspring, conditional on the parents' genome. The expectation here is over the potential offspring with higher‐ranked individuals.

We consider minimizing the expected kinship (*K*), which we compute not from pedigrees (which we call theoretical kinship) but conditional on the local genome (which we denote empirical kinship). Specifically, **kinship** *K* is defined as the average proportion of the genome that is shared from the same founder haplotype (mathematically defined in Section 4.1). We also maximize expected heterozygosity (*H*; Section 4.2), which is defined as the proportion of the genome that contains a heterozygous (i.e. different) allele. We contrast this with minimizing expected introgressed admixture (*Q*; Section 4.3), defined as the average proportion of the genome that is from the introgressing population.

**FIGURE 3:**
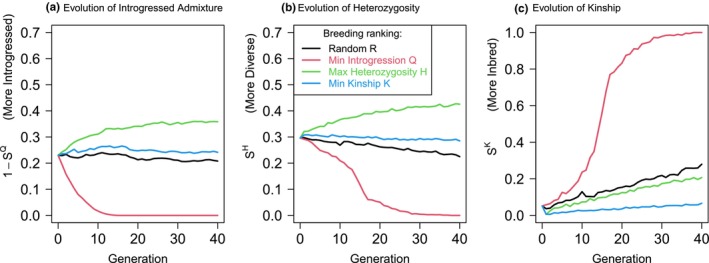
Simple measures cannot simultaneously remove introgressed ancestry and maintain diversity. In this simulated captive population, we either breed random pairs (R), attempt to minimize kinship (K) or introgression (Q), or to maximize heterozygosity (H). We quantify their performance over time with scores for (a) introgressed admixture fraction $1-S^{Q}$, (b) heterozygosity *S* ^*H*^ and (c) kinship *S* ^*K*^.

A successful program must reduce introgression without a large cost in either kinship or heterozygosity. This is scored in the realized population using the same measures that are used as a selection measure. When used as a score, these are denoted as *S* ^*Q*^ for admixture, *S* ^*H*^ for heterozygosity, and *S* ^*K*^ for kinship. Scores are distinct from expected measures because they are observed in the current population, not an average over possible pairs. Figure 3 shows results for a single simulation which are confirmed to be representative below. No measure is satisfactory: minimizing introgressed admixture leads to a diversity collapse (both for heterozygosity and kinship). Conversely, minimizing kinship best maintains the population as it was, whilst maximizing heterozygosity increases introgression as the introgressing population is diverged from the target.

Local ancestry information allows us to assign ancestry to specific genomic loci. In order to balance population ancestry and diversity, we introduce population heterozygosity (PH, Section 4.4) and population kinship (PK, Section 4.5). These are again defined as the average proportion of genome that is heterozygous or inherited from the same founder, but with variants that are not inherited from the target population being ‘masked out’, that is, treated as heterozygosity 0 and kinship 1. These measures exploit being able to assign each specific position on a genome to a population and can be interpreted as the mean value (of *H* or *K*) at loci on haplotypes where *both* copies were inherited from the target population. Population heterozygosity and population kinship are easily evaluated as scores *S* ^*PH*^ and *S* ^*PK*^ respectively, averaged across realized individuals.

The use of population measures punish hypothetical offspring with one or two introgressed haplotypes equally. To address this, we also consider weighted population heterozygosity (WPH) in Section 4.6 and weighted population kinship (WPK) in Section 4.7, in which a weight δ is placed on the population measure (kinship or heterozygosity) and 1−δ is placed on expected admixture *Q*. Throughout we use δ=0.5 which for heterozygosity has the appealing interpretation of placing relative weight 0 on two introgressed alleles, 1 on a single target population allele, 2 on homozygous target population alleles and 4 on heterozygous target population alleles.

**FIGURE 4:**
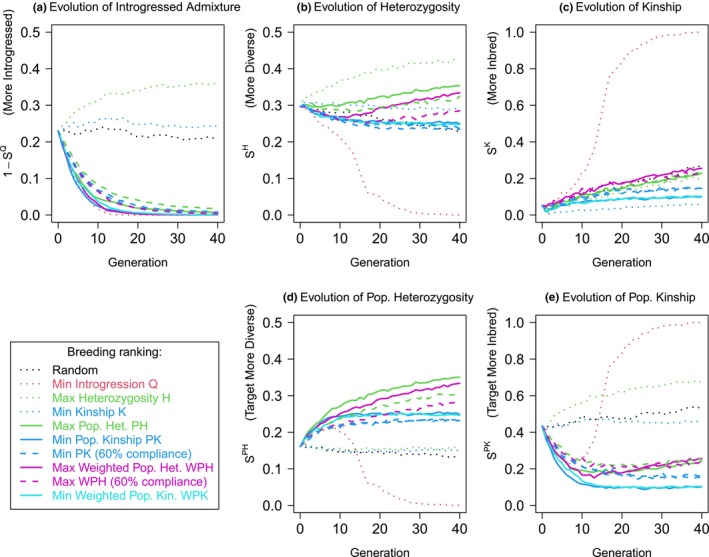
Local ancestry‐based measures can simultaneously remove introgressed ancestry and maintain diversity. This is the same simulation as Figure 3, which is shown as dotted curves, and we add curves attempting to minimize population kinship, population heterozygosity, and their weighted versions. We also add ‘60% compliance’ curves for which the recommendation is followed 60% of the time, and a random choice of parent is made otherwise. We quantify performance over time with scores for (a) introgressed admixture fraction $1-S^{Q}$, (b) heterozygosity *S* ^*H*^, (c) kinship *S* ^*K*^, (d) population heterozygosity *S* ^*PH*^ and (e) population kinship *S* ^*PK*^.

The result of using each of these local ancestry measures are shown in Figure 4. All suggested local ancestry measures control introgression nearly as well as minimizing it directly. Simultaneously, they maintain heterozygosity and kinship adequately overall, and strongly improve them for genetic material from the conservation target population. To minimize kinship, both weighted and unweighted population kinship measures perform similarly. Weighted population heterozygosity could be argued as preferable as it maintains heterozygosity above the starting value. Importantly, and perhaps surprisingly, if we allow the realities of the breeding program to allow only 60% of breeding recommendations to be followed (60% ‘compliance’; Section 4.10.2), with other parents chosen randomly, the recovery is still dramatic with strong control over admixture *Q* and no severe consequences for heterozygosity or kinship.

### 2.3 Sensitivity analysis

**FIGURE 5:**
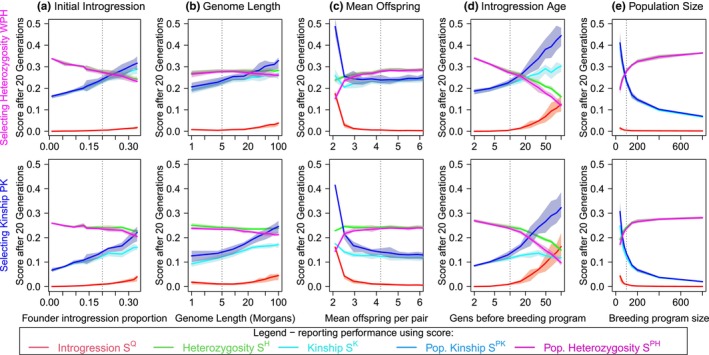
Impact of the simulation parameters on the scores after 20 generations of selective breeding using (top) the weighted population heterogeneity (WPH) measure and (bottom) population kinship (PK). This is in populations with otherwise the same demography as Figures 3 and 4 (whose parameters are shown as vertical line). Reported is the mean and 25%–75% quantile of 10 simulations per parameter value. Here we consider changing (a) the initial amount of introgression observed in the founder captive population, (b) the total quantity of genome in Morgans, retaining the same number of SNPs, (c) the average number of offspring who survive to adulthood and are available for the next generation of breeding program, (d) the number of generations between admixture and the creation of the breeding program and (e) the number of individuals in the breeding program. The scores are the five shown as separate panels in Figure 4, colour‐coded to their corresponding breeding measures.

Having established that the genomic measures work in principle, we now more thoroughly consider the details of the simulation scenario, which can affect the speed that the breeding program operates and the final outcome in terms of diversity. We focus on the best two options, selecting for population kinship (PK) or weighted population heterozygosity (WPH), which perform quite similarly. Now reporting distributions over 10 simulations per parameter combination, Figure 5 considers several alternate scenarios. Focus should be placed on the final introgression level (red curve), with the kinship and heterozygosity (cyan and green) and their population counterparts (blue and purple) being reported to show the diversity costs.

Figure 5a shows that heterozygosity decreases with initial introgression in proportion to the diversity of target ancestry present in the original panel. Kinship likewise increases due to fewer founders being available at each locus. The population‐specific scores closely match the introgression‐free ones, and only deviate when there is limited power to remove introgression over the timeframe (here 20 generations). Both selection options are adequate, with heterozygosity selection increasing both kinship and heterozygosity slightly.

Figure 5b shows that increasing the genome length decreases the selection efficiency, with a corresponding increase in introgression *S* ^*Q*^. Kinship, heterozygosity and population heterozygosity scores are not strongly affected. Population kinship is increased for longer genomes. We hypothesise that this is due to having more introgressed segments remaining, and a decreased diversity of candidates as time progresses, meaning that replacing introgressed segments later comes at a higher kinship cost. Animal conservation target species are likely to have less than 50 M (Morgans) of genome, with sex‐averaged genetic map lengths being surprisingly consistent across genera; for example, the mouse (16.3 M (Dreau et al., 2019)), cat (43.7 M), superb fairy‐wren (16.2 M (Penalba et al., 2020)) and stickleback (12.5 M (Dreau et al., 2019)) genomes are all on the same order of recombination length to humans (35 M (Kong et al., 2002)), and in all cases genome length is not a barrier to removing introgression after 20 generations. Larger brood sizes typically lead to more options for breeding and therefore a more efficient breeding program. Figure 5c shows that the process works well provided that the number of offspring who themselves survive to breeding age is above around 3.

One of the more dramatic changes to a scenario is the number of generations before admixture. Focusing on the red curve (the introgression score) of Figure 5d highlights how vital it is to make an early intervention: after about 10 generations, the ability to reduce introgression decreases, and the resulting cost in kinship is increasingly high. This is because the introgressed DNA exists in increasingly small segments as the introgression event fades into the past. We emphasize that the initial introgression level was controlled to 25%, and that we are examining the population after 20 generations of the breeding program. Breeding for longer can offset a delay — in Figure S2 we vary the delay and breeding program duration, and measure introgression. A program running the same duration as the introgression age will remove most of the introgressed DNA (down to 2%), but the time taken to completely eliminate introgression grows. Selecting for population kinship seems preferable to selecting for population heterozygosity as the age increases. Noting the increased Kinship cost of WPH over PK in Figure 5d, we hypothesise that minimizing kinship better protects haplotype diversity in the population, and thus provides more selection options in the future. Finally, we assume here that the number of individuals is kept constant at historic levels, but Figure 5e shows that a larger breeding program does improve efficiency, with the primary gain predictably being in kinship and heterozygosity.

### 2.4 Application to the Scottish wildcat

To predict how effective this procedure could be on real‐world introgression patterns, we apply the breeding simulation forward in time to the best approximation of the current Scottish wildcat breeding population that can be achieved with current data. We emphasize that these data, obtained to evaluate the history of wildcat introgression in Scotland, are considerably more introgressed than the breeding program individuals, due to containing a high number of wild‐living individuals. The following information is given in more detail where the data were first presented (Howard‐McCombe et al., 2023; Jamieson et al., 2023).

We start with a dataset of 36 cats (Howard‐McCombe et al., 2023) consisting of 6 captive cats in the Scottish wildcat breeding program, plus 30 wild living cats collected as part of a diversity panel (i.e. individuals were chosen to be widely geographically distributed and representing diverse populations). Introgression was confirmed in the captive population using ancient DNA as well as historical museum samples. The captive cats had an average domestic cat introgression of 0.18, whereas the wild‐living cats ranged from 0.11 to 0.82. To replicate a genomic panel that we might find in the captive population, we annotated each of these individuals as founders, selected the top 24 by genome‐wide wildcat genome proportion (maximum 0.53, mean 0.18) and performed a single breeding step to create a ‘captive population’ of *N* = 150 individuals.

The whole cat genome is 43.7 Morgans (Li, Hillier, et al., 2016), and we analyse chromosome E3 which consists of 1.26 Morgans observed at 17720 SNPs. Because the introgression is relatively recent, the chromosome varies considerably from genome‐wide admixture fractions, with a mean wildcat ancestry per haplotype of 0.60 and a range of 0.14–1.00. We applied realistic choices for simulation parameters (number of lifetime offspring per pair; carrying capacity) matched to the breeding program (Section 4.11).

**FIGURE 6:**
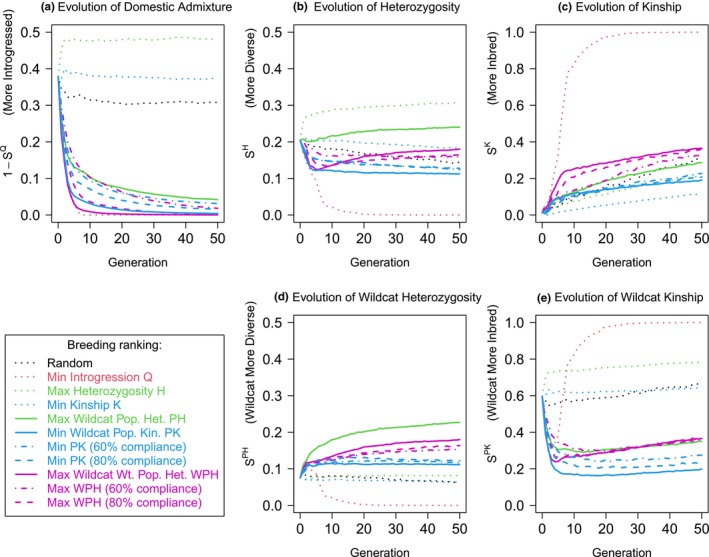
Results for a putative Scottish wildcat breeding program based on real genetic data and local admixture estimation for 28 founders and a captive population capacity of 150. Curves are as Figure 4, showing traditional measures as dotted lines and best‐case local ancestry measures as solid lines. ‘Real world’ limited compliance (80% dashed, 60% dot‐dashed) with breeding recommendations are also shown. The scores are (a) domestic cat admixture fraction $1-S^{Q}$ (note that these founders have higher introgression that the captive breeding program); (b) heterozygosity *S* ^*H*^, (c) kinship *S* ^*K*^, (d) wildcat population heterozygosity *S* ^*PH*^, and (e) wildcat population kinship *S* ^*PK*^.

Figure 6 shows the results of the same breeding process described for the simulated data for the case of Scottish wildcat introgression. Parameters have been matched to the breeding program where possible: the mean number of offspring per breeding pair is 4.2, which is the lifetime expected number per female as observed in the stud book (Barclay, 2019) and 150 individuals is the breeding program size. Qualitatively the results agree with results on the simulated haplotypes, with the local‐ancestry measures successfully controlling introgression and maintaining diversity. Due to the lower number of founders and the higher initial introgression, there is a separation between the scores, with WPH removing introgression significantly faster than selection for the PK kinship measure (especially over a 5–10 generation horizon) but at the price of increased kinship. In this case, the ability to comply with breeding recommendations does start to have an impact; an 80% compliance rate is qualitatively similar to full compliance and can achieve eventual wildcat ancestry recovery, whereas a 60% compliance rate leads to considerably slower recovery.

## 3 DISCUSSION

### 3.1 Pathway to deployment and constraints

When deploying to a conservation program, local ancestry is not observed and must be inferred (Figure 1 left). This leads to all the regular issues associated with bioinformatics and local ancestry inference, which we outline for readers unfamiliar with the process. During program setup the genomic framework must be established. The reference data and first generation breeding population will need medium coverage sequencing or high density SNP arrays (this varies by species, but 500‐k is advised to contain enough LD to estimate haplotypes). A reference genome assembly from a related species is essential as we can only conserve genetic material that has been mapped to a reference. Limited genomic rearrangements (i.e. structural changes between the reference and conservation target species) or the use partially assembled genome contigs should be expected to work with a minor impact on local ancestry inference. The number and diversity of reference assemblies is growing rapidly (Hotaling et al., 2021) and where it is absent, it may be constructed *de‐novo* (Baker, 2012) (i.e. from long‐read sequence data alone), and trio data (parents plus child) from a breeding program is of particular help (Koren et al., 2018). For what follows we therefore assume that the data start as a quality‐controlled Variant Call Format (VCF) file. A genetic map (i.e. the expected recombination rate per basepair (Peñalba & Wolf, 2020)) will improve accuracy of local ancestry inference, though adequate local ancestry inference should be possible assuming a uniform rate of recombination.

The first stage of analysis for any generation of the program is statistical phasing, that is learning which genomic segments were co‐inherited from each parent. Phasing is straightforward, particularly where parental genomes are available, and can be achieved with for example the software BEAGLE (Browning & Browning, 2007) (v5). The same software can also perform imputation, that is, inferring any missing variants, by comparison to reference data (here, including both species and ancestors), which allows more affordable lower‐coverage sequencing. For example, even 1X coverage (costing as little as $50 for a 5 Billion base genome in 2022 according to NIHR) in 50b segments will sample the two copies of a 100 kb haplotype 1 k times, which may adequately identify which haplotype was inherited (Vieira et al., 2016).

In the second stage, we compare the haplotypes to the reference datasets to infer which population each genomic segment was inherited from. This can be achieved with high accuracy using reference data consisting of a diverse panel of representative individuals from both the introgressing and target species. Some local ancestry inference software such as MOSAIC (Salter‐Townshend & Myers, 2018) allows these to be slightly introgressed, but careful curation of this data is very important. Local ancestry inference works best if the introgressing species is highly diverged from the target species, which is measured in terms of *F*_*ST*_ as described above. An *F*_*ST*_ of 0.4 as observed between domestic and wildcat is more than large enough; there is no requirement that any sites are segregating in only one species.

Finally, we need to compute the selection measures. Computing (weighted) population heterozygosity is straightforward from genetic data, but estimating kinship from the local genome is only direct if the founders were sequenced. While estimating founders' genomes is possible (Finke et al., 2021), a kinship measure based on observable segments of IBD would be simpler. Thus the initialization of a program is a bioinformatics‐heavy challenge, but the long‐term process can (in principle) be automated and managed by conservationists. For this, software will be needed that incorporates these genomic features while maintaining all of the small subtle details that allow the simulation to represent a breeding program, including the correct generational structure, the practicalities of creating mating pairs, etc, integrating bioinformatics into a tool resembling PMx (Ivy & Lacy, 2012).

Beyond the implementation requirements described above, many challenges remain. A primary one is maintaining funding. While the cost of sequencing continues to drop, obtaining high‐density genetic information for a minimum of 100 individuals over 10 generations is a considerable burden to maintain over time. Another is the ticking clock of diversity within species: as captive populations are maintained in small populations, kinship inevitably increases. The health consequences of kinship, or inadvertent selection within a breeding program, as measured this way are unclear. At least in our simulated breeding conditions we were able to maintain heterozygosity even in the face of rising kinship, which is present in increasingly small segments of Identity by Descent. While there are no absolute barriers to deployment for any recently introgressed species, the initial bioinformatics and reference population dataset construction is costly, and the skills to do this are not widely distributed in conservation programs. Further, if non‐introgressed individuals from the target population are not available (from ancient DNA, museum samples, other populations, etc.) then recovery depends on statistical inference which needs careful testing.

### 3.2 Implications for de‐introgression

We have shown that traditional measures used for breeding decisions in captive breeding programs are inadequate for populations that experienced introgression prior to the initialisation of the captive population. For such populations we must be very careful not to mistake introgression for diversity. However, a variety of measures based on using ‘local ancestry’ to determine the introgression status of each genomic region are extremely effective at reducing introgression while simultaneously maintaining genetic diversity, whether measured in terms of kinship or heterozygosity.

For the case of the Scottish wildcat, we have shown that the additional information required, that is local ancestry information, is quite straightforward to access in practice (Howard‐McCombe et al., 2023). From relatively low‐coverage whole genome sequencing data we would be able to accurately infer haplotypes via statistical phasing, and attribute local ancestry. As a pilot demonstration, we were able to perform the breeding program *in‐silico*, not from the active captive population (for which sequencing is unavailable) but using a diversity panel. Simply by sequencing the active breeding population of a program, we could apply our pipeline to improve breeding pair selection.

A critical component of a successful reversal of hybridization is timing. It is only possible to undo recent hybridization, as the required duration of the breeding program will grow in proportion to the delay, which places strong practical and cost constraints on the process. ‘Unscrambling the egg’ of hybridization takes as long as it was left to scramble so there are considerable cost savings and practicality benefits in acting early, if the decision is to act at all. For the case of the Scottish wildcat diversity panel data, estimates for individuals range from between 4.7 and 17.9 generations before present, with a mean estimate of 8.6 (95% CI 8.3–9.8) generations. This scale of hybridization is consistent with a predicted potential recovery over 10–20 generations of breeding program, provided that compliance with the breeding recommendations is not too low. Without sequencing the captive cats, we can only say that the option is promising; the ‘future captive breeding population’ simulated here differs in both the initial admixture and diversity, the genome length simulated, and in the particulars of generation structure. These particulars are required to tell how stringently breeding recommendations must be followed to achieve adequate recovery in practice.

### 3.3 Further opportunities

Some genomic features of captive populations may require special treatment. We have not modelled inbreeding depression beyond a simple increased mortality in the first year. In modelling tools such as PMx (Ivy & Lacy, 2012) in which the genome is not available, recessive disorders are modelled as independent random events occurring with rate proportional to the kinship coefficient. We have the advantage of observing the kinship for specific loci, which might in principle be used to infer regions of increased and decreased inbreeding risk. Similarly, outbreeding depression may not be uniform along the genome. This can in principle be learnt and modelled.

Non‐introgressed species facing extreme small population size (Bosse & van Loon, 2022) face deleterious or even lethal recessive disorders, with two examples being chondrodystrophy in California condor (*Gymnogyps californianus*) (Ralls et al., 2000) and blindness in red‐billed choughs (*Pyrrhocorax pyrrhocorax*) (Trask et al., 2016). Our study implies that direct genetic manipulation (Johnsson et al., 2019) can be contrasted with deliberate genomic rescue through hybridization. The desired specific variants can be retained whilst unwanted introgression is subsequently removed. This can be implemented by relaxing the preference for ancestry at targeted genetic loci.

Immune genes may be considered similarly. For example, the major histocompatibility complex (MHC) region which regulates immune response, may have been selected for domestic cat ancestry in wildcats (Howard‐McCombe et al., 2023), due to the high domestic cat disease burden. Breeding programs may wish to take this into account. This is related to the desired outcome – how much introgression needs to be removed? The answer may well be related to real‐world measures of both fitness and ecological function, which are yet to be robustly related to genomic properties. It is possible that widespread linkage of pedigrees with phenotypes and genomic quantification of populations over time can address these problems, but that is yet to be proven.

The potential that such fine‐scale genomic information brings to conservation genetics implies that a new generation of tools may use far more detailed representation of the genome for selection than the simple scores defined here. Some caution may therefore be needed before the tools are mature, but the advantages of acting early to save the diversity that is present needs to be weighed against the risks.

## 4 METHODS APPENDIX

In this section, we provide precise mathematical details of the information that is described in Section 2. The model is currently a population‐based model but could be extended to have Individual‐based components for more detailed predictions. Throughout we will follow at generation time *t* a genetic panel *G*(*t*) making up a 3D array of dimension $N\left(t\right)\times 2\times L$. $G_{\mathrm{ial}}\left(t\right)$ is indexed by the individual $i=1\ldots N\left(t\right)$, the haplotype copy *a* = 1,2 and the genetic loci l=1…L, and takes value 0 for reference (i.e. no mutation) and 1 for alternate (i.e. mutation) alleles. We will also follow an ancestry panel *A*(*t*) of $N\left(t\right)\times 2\times L$ local ancestry values, taking value 1 for our Target ancestry and 0 for Introgressed ancestry. To ease the notation, where possible we omit the time variable when referring to the current generation. We also keep a simple population size schedule where $N\left(0\right)=N_{F}$ is the number of founders and the captive population size $N\left(t\right)=N$ is fixed for *t* > 0.

*G*(*t*) and *A*(*t*) are tracked directly from one generation to the next using the process of meiosis (see Section 4.8).

### 4.1 Kinship coefficient and local pedigree

For the simulation study, we will follow the ‘local pedigree’ or founder panel *F*(*t*) of $N\left(t\right)\times 2\times L$ founder sources, describing the founder haplotype of each genomic locus *l* from a founder population forwards in time. For individual *i*, *F* is initialised to $F_{\mathrm{ial}}\left(0\right)=2i-I\left(a=1\right)$ with $a\in \left(1,2\right)$, that is the whole of each haplotype in the founder population is assigned a unique value from 1 to $2N_{F}$. *I*(*x*) is an indicator function, which for binary *x* takes value 0 when *x* is false and 1 when *x* true. Like *G* and *A*, *F* is tracked during meiosis (Section 4.8).

By tracking the founder panel we can compute the (empirical) expected kinship coefficient *K*_*ij*_ between individuals *i* and *j*. The kinship coefficient is the probability that two randomly chosen alleles are IBD and is typically conditioned on the pedigree alone (which we call the theoretical kinship). However, as we observe the genome it is useful to condition on it (which we call the empirical kinship or simply the kinship). The expectation is taken over potential offspring and is the average over each specific locus *l*. The *expected kinship coefficient* $K_{\mathrm{ij}}=\sum_{l=1}^{L}K_{\mathrm{ijl}}$ is to be written in terms of $F_{\mathrm{ial}}$, the founder ancestor of haplotype *a* from individual *i* at locus *l*:

$$K_{\mathrm{ijl}}=\frac{1}{4}\sum_{a\in \left(1,2\right)}\sum_{b\in \left(1,2\right)}I\left(F_{\mathrm{ial}}=F_{\mathrm{jbl}}\right).$$

Although $F_{\mathrm{ial}}$ is not directly estimable from data, the IBD status $I\left(F_{\mathrm{ial}}=F_{\mathrm{jbl}}\right)$ is (Ivy et al., 2016; Speed & Balding, 2015), so *K* can still be estimated at additional computational cost.

The expected kinship coefficient *K*_*ij*_ is used for decision making. The score that we report for performance is the empirical mean for realized individuals, that is the ‘kinship score’ is $S^{K}=\frac{1}{N}\sum_{i=1}^{N}S_{i}^{K}$ where the individual kinship is:

$$S_{i}^{K}=\frac{1}{L}\sum_{l=1}^{L}I\left(F_{i1l}=F_{i2l}\right).$$

Kinship is to be minimized. Throughout we define expected values for pairing for decisions as matrices using capital letters and scores based on them as superscripts on *S*, that is *K* and *S* ^*K*^.

### 4.2 Heterozygosity

Analogously to kinship, we can define heterozygosity as both a score and a pairwise expectation for the offspring of each pair of individuals. Given a pair of individuals *i* and *j*, the *expected heterozygosity* of their offspring is $H_{\mathrm{ij}}=\frac{1}{L}\sum_{l=1}^{L}H_{\mathrm{ijl}}$ where for locus *l*:

$$H_{\mathrm{ijl}}=\frac{1}{4}\sum_{a\in \left(1,2\right)}\sum_{b\in \left(1,2\right)}I\left(G_{\mathrm{ial}}\neq G_{\mathrm{jbl}}\right).$$

The heterozygosity score $S^{H}=\frac{1}{N}\sum_{i=1}^{N}S_{i}^{H}$ where $S_{i}^{H}=\frac{1}{L}I\left(G_{i1l}-G_{i2l}\right)$, that is takes value 0 if the two copies are the same and 1 otherwise. Heterozygosity is to be maximized.

### 4.3 Admixture

It is natural to quantify ancestry via the proportion of ancestry from the target population, by convention called the ‘Q’ statistic. We have defined $A_{\mathrm{ial}}=1$ if the *a*‐th haploptype from individual *i* at locus *l* is from our target population.

The *expected admixture* is $Q_{\mathrm{ij}}=\frac{1}{L}\sum_{l=1}^{L}Q_{\mathrm{ijl}}$ where

$$Q_{\mathrm{ijl}}=\frac{1}{8}\sum_{a\in \left(1,2\right)}\sum_{b\in \left(1,2\right)}\left(A_{\mathrm{ial}}+A_{\mathrm{jbl}}\right).$$

The admixture score is $S^{Q}=\frac{1}{N}\sum_{i=1}^{N}S_{i}^{Q}$ where $S_{i}^{Q}=\frac{1}{2L}\left(A_{i1l}+A_{i2l}\right)$ and is to be maximized. We will report in terms of the introgression score $1-S_{i}^{Q}$ which is to be minimized.

### 4.4 Population heterozygosity

We now condition on local ancestry to make a population‐specific score. This is slightly more transparent for heterozygosity than for kinship, and for the score rather than the expected value in the next generation, though both follow analogously. Population heterozgosity (PH) is a product of two terms, whether both copies come from the target population, and whether the SNP is heterozygous conditional on this. We define the target ancestry diploidy $I\left(A_{1l}+A_{2l}=2\right)$ as an indicator function taking value 1 if both haplotypes are derived from the target population, or 0 otherwise.

The *expected population heterozygosity* $PH_{\mathrm{ij}}=\frac{1}{L}\sum_{l=1}^{L}PH_{\mathrm{ijl}}$ is defined for a pair of individuals *i* and *j* and the expectation is taken over their potential offspring:

$$PH_{\mathrm{ijl}}=\frac{1}{4}\sum_{a\in \left(1,2\right)}\sum_{b\in \left(1,2\right)}I\left(A_{\mathrm{ial}}+A_{\mathrm{jbl}}=2\right)I\left(G_{\mathrm{ial}}\neq G_{\mathrm{jbl}}\right).$$

The PH score $S^{\mathrm{PH}}=\frac{1}{N}\sum_{i=1}^{N}S_{i}^{\mathrm{PH}}$ is the average over realized individuals' scores $S_{i}^{\mathrm{PH}}=\frac{1}{L}\sum_{l=1}^{L}S_{\mathrm{il}}^{\mathrm{PH}}$ across the genome, where:

(1) $$S_{\mathrm{il}}^{\mathrm{PH}}=I\left(A_{i1l}+A_{i2l}=2\right)I\left(G_{i1l}\neq G_{i2l}\right).$$

Population heterozygosity is to be maximized. It is equal to the heterozygosity if all copies of all loci are from the target population.

### 4.5 Population kinship

The population kinship is constructed as the population heterozygosity, but more care is needed to ensure that the target population ancestry is selected to increase whilst kinship is selected to decrease. We wish to only reward loci that both are from the target ancestry and have different founders $I\left(F_{\mathrm{ial}}\neq F_{\mathrm{jbl}}\right)$. The *expected population kinship* $PK_{\mathrm{ij}}=\frac{1}{L}\sum_{l=1}^{L}PK_{\mathrm{ijl}}$ is therefore defined as:

$$PK_{\mathrm{ijl}}=1-\frac{1}{4}\sum_{a\in \left(1,2\right)}\sum_{b\in \left(1,2\right)}I\left(A_{\mathrm{ial}}+A_{\mathrm{jbl}}=2\right)I\left(F_{\mathrm{ial}}\neq F_{\mathrm{jbl}}\right).$$

Similarly, the *population kinship score* $S^{\mathrm{PK}}=\frac{1}{N}\sum_{i=1}^{N}S_{i}^{\mathrm{PK}}$ is the sum over the individual realized population scores $S_{i}^{\mathrm{PK}}=\frac{1}{L}\sum_{l=1}^{L}S_{\mathrm{il}}^{\mathrm{PK}}$ where the per‐locus score is:

$$S_{\mathrm{il}}^{\mathrm{PK}}=1‐I\left(A_{i1l}+A_{i2l}=2\right)I\left(F_{i1l}\neq F_{i2l}\right).$$

Population kinship is to be minimized and is equal to the kinship if all copies of all loci are from the target population.

### 4.6 Weighted population heterozygosity

There is no reason to expect that the best‐performing rule should only consider ancestry homozygotes for the target population – particularly for rare SNPs, it might instead be better to reward any ancestry. We therefore consider the *weighted population heterozygosity* $\mathrm{WP}H_{\mathrm{ij}}=\frac{1}{L}\sum_{l=1}^{L}\mathrm{WP}H_{\mathrm{ijl}}$ where:

$$\mathrm{WP}H_{\mathrm{ijl}}=\frac{1}{8}\sum_{a\in \left(1,2\right)}\sum_{b\in \left(1,2\right)}\left(1-\delta \right)\left(A_{\mathrm{ial}}+A_{\mathrm{jbl}}\right)+2\mathrm{\delta I}\left(A_{\mathrm{ial}}+A_{\mathrm{jbl}}=2\right)I\left(G_{\mathrm{ial}}\neq G_{\mathrm{jbl}}\right).$$

WPH is to be maximized. If we set δ=0 this returns to the target population admixture $Q_{\mathrm{ijl}}$, and with δ=1 this becomes the population heterozygosity. We use δ=0.5 throughout, which rewards heterozygosity equally to ancestry dosage, and which leads to the per‐locus value 0 for no target alleles, 0.25 for a single target allele, 0.5 for two identical target alleles, and 1 for heterozygous target variants.

### 4.7 Weighted population kinship

The same procedure can be applied to obtain the *weighted population kinship* $\mathrm{WP}K_{\mathrm{ij}}=\frac{1}{L}\sum_{l=1}^{L}\mathrm{WP}K_{\mathrm{ijl}}$ where:

$$\mathrm{WP}K_{\mathrm{ijl}}=\frac{1}{8}\sum_{a\in \left(1,2\right)}\sum_{b\in \left(1,2\right)}\left(1-\delta \right)\left(2-A_{\mathrm{ial}}-A_{\mathrm{jbl}}\right)+2\delta \left(1-I\left(A_{\mathrm{ial}}+A_{\mathrm{jbl}}=2\right)I\left(F_{\mathrm{ial}}\neq F_{\mathrm{jbl}}\right)\right).$$

This transitions between selection against introgression and selection against kinship. WPK is to be minimized.

### 4.8 Simulated meiosis

Individual genomes are observed over a single chromosome with genome length *D* (measured in Morgans, default 5) at *L* single nucleotide polymorphisms (SNPs; default 2000). *D* is the average number of recombination events separating parent haplotypes from the offspring haplotype. Let *R*_*l*_ for l=1,…,L denote the recombination position of the *l*‐th SNP, that is taking values in the range $\left(0,D\right)$. In the simulation we evenly‐space SNPs so that SNP *l* has recombination position $R_{l}=\mathrm{lD}/L$.

Meiosis begins by producing a gamete for each parent. Let $a\left(l=1,\ldots ,L\right)$ be a vector of length *L* taking value 1 or 2 to denote which of the two haplotypes is being inherited at position *l*. We generate *a* via an independent crossover recombination process:

1. Initialise:
  - Set the initial position $p\left(b=1\right)=0$ where *b* indexes the recombination segments, starting at *b* = 1.
  - Choose an initial haplotype $h\left(b=1\right)∼U\left(1,2\right)$, that is start with either the first or second haplotype with equal probability.
2. Until $p\left(b\right)>R_{L}$, that is the whole chromosome is processed:
  - Generate a segment inheritance length $l\left(b\right)∼\exp\left(1\right)$.
  - Let $e\left(b\right)=p\left(b\right)+l\left(b\right)$ be the end point of the inherited segment. For every SNP in the segment, that is for *l* such that $p\left(b\right)\leq R_{l}<e\left(b\right)$, set $a\left(l\right)=h\left(b\right)$.
  - Set $h\left(b+1\right)=3-h\left(b\right)$, that is switch from haplotype 1 to 2 and vice‐versa. Set the next start point to be the current end point, that is $p\left(b+1\right)=e\left(b\right)$, and move on to the next segment setting b←b+1.

We generate the gamete *a*_*i*_ for parent *i* and *a*_*j*_ for parent *j*, tracking *G*, *A* and *F*. Specifically for the *k*‐th offspring in generation *t* + 1 with parents *i* and *j*, we set $G_{k1l}\left(t+1\right)=G_{ia_{i}\left(l\right)l}\left(t\right)$ and $G_{k2l}\left(t+1\right)=G_{ja_{j}\left(l\right)l}\left(t\right)$, and likewise for *A* and *F*.

### 4.9 Simulated population

The simulation uses two related populations *P*_*A*_ and *P*_*I*_, the target and introgressing populations respectively, who share ancestry via a common ancestor *P*_0_. The common ancestor has SNPs l=1…L taking value 0 by default or 1 with frequency $f_{l}∼U\left(0.05,0.95\right)$ and the population frequencies follow the Balding‐Nichols model (Balding & Nichols, 1995) with *Fst F*_*A*_ and *F*_*I*_ respectively (both assumed 0.2):

$$f_{\mathrm{Al}}∼\text{Beta}\left(f_{l}\frac{1-F_{A}}{F_{A}},\left(1-f_{l}\right)\frac{1-F_{A}}{F_{A}}\right),$$

with expectation *E*(*f*_*Al*_) = *f*_*l*_ and variance $\mathrm{Var}\left(f_{\mathrm{Al}}\right)=F_{A}f_{l}\left(1-f_{l}\right)$. The same model is applied to the introgressing population *P*_*I*_. The genetic separation between populations *P*_*A*_ and *P*_*I*_ is by construction $F_{\mathrm{ST}}=F_{A}+F_{B}=0.4$.

We form an introgressed *wild* population as follows. First we generate a genetic panel *G*_*A*_ of 2*N*_*A*_ haplotypes to form the pre‐introgression target population, matched with an ancestry panel *A*_*A*_ with every entry taking value 1. We also form an introgressing panel *G*_*I*_ of 2*N*_*I*_ haplotypes to form the introgressing population, matched with *A*_*I*_ with every entry taking value 0. We then impose a sequence of introgression rates $\alpha \left(1\right),\ldots ,\alpha \left(T_{\text{init}}\right)$, using $T_{\text{init}}=10$ and $\alpha \left(1,2,3,\ldots ,10\right)=\left(0.2,0.2,0,\ldots ,0\right)$, that is introgression happened 10 generations in the past and lasted 2 generations. We then perform random mating with the specified introgression rate. Random mating uses the Wright‐Fisher process, that is each offspring chooses two random parents, with probability $\alpha \left(t\right)$ uniformly from the introgressed panel and with probability $1-\alpha \left(t\right)$ from the target panel. The offspring are generated by the meiosis process described in Section 4.8.

We then perform two additional generations to form a more realistic *captive* population: a *bottleneck* to replicate a small number of founder individuals being sampled for introduction into the program, and *selection* for the most representative of those founders. In the bottleneck step, a fraction *p*_bottleneck_ (=0.25 here) of individuals are sampled for uniform random breeding. These individuals are indexed to define the founders. To form the most promising breeding population, in the captive step, a fraction *p*_captive_ (=0.25 here) are selected by minimum introgression proportion and finally bred (using the Wright‐Fisher process) to form the *captive* population containing *N* individuals. Introgression therefore occurred $T_{\text{init}}+2$ generations ago.

### 4.10 Simulated breeding program

Here we describe how captive individuals are chosen for breeding within the captive breeding program. A breeding program contains many details that will differ from a simulation. Our conclusions are robust to the particular design of the generational structure (with sensitivity analyses described below.) For ease of exposition we focus on a discrete‐generation process in which a random number of offspring are produced per breeding pair $n_{\mathrm{ij}}∼F_{n}=\text{Poisson}\left(\lambda \right)$ (with mean 1/λ=4.2 here).

#### 4.10.1 Ranked breeding rule

Breeding programs can use a range of schemes to pair individuals (Ivy & Lacy, 2012) based on minimizing (or maximizing) a breeding score *B*_*i*_. The breeding score should not be considered as a property of an individual, but is instead based on a pairwise matrix *B*_*ij*_ that can be a function of the next generation being constructed. The expected (i.e. mean) kinship $B_{i}^{\mathrm{MK}}=\frac{1}{n_{i}}\sum_{j}K_{\mathrm{ij}}$ is a popular choice, where the expectation is taken over *n*_*i*_ individuals determined by a selection process (see below). The expected admixture *Q*_*i*_, inbreeding coefficient, identity‐by‐state, identity‐by‐descent, or similar can all be used. (Ivy & Lacy, 2012) describe processes based on ranking, with elimination of pairs that are too genetically similar. For discrete generations they found that ‘ranked selection’ was appropriate. We implement their scheme without sex constraints for simplicity. This creates a ‘breeding score’ *B*_*i*_ for each individual, that is updated to reflect the other potential parents expected to be in the next generation. This is a good approach because the breeding scores computed can be seen as expected values over the entire captive population and hence reflect the ‘value’ of the individual to the whole breeding program.

The procedure is as follows (assuming we are maximizing *B*):

1. Compute *B*_*ij*_ for the current generation.
2. Set the parent stack *s* = () empty, and the remaining list $r=\left(1,\ldots ,N\right)$ containing all individuals.
3. Iteratively construct the parent stack, from least promising to most promising. For *t* in 1,…,N:
  - Compute the breeding score $B_{i}=\frac{1}{|r\left(t\right)|}\sum_{j\in r\left(i\right)}B_{\mathrm{ij}}$ which is an average breeding score over the $|r\left(t\right)|$ remaining individuals;
  - Select the worst individual *k* = argmin_*i∈r*_ *B*_i_;
  - Add *k* to the parent stack, that is set $s=\left(s,k\right)$;
  - Remove *k* from the remaining list, that is set *r* = (*r*\*k*).
4. Perform pairing from most promising to least promising. Set pair number *t* = 1, current offspring count $n\left(1\right)=0$. Then while $n\left(t\right)<N$:
  - Choose parent 1 as the best $p_{1}=s\left(N-t+1\right)$ and parent 2 as the next best $p_{2}=s\left(N-t\right)$ remaining in the stack;
  - Add $n_{p_{1},p_{2}}∼F_{n}$ offspring individuals to the next generation via meiosis from their parents $\left(p_{1},p_{2}\right)$. Set $n\left(t+1\right)=n\left(t\right)+n_{p_{1},p_{2}}$.
  - Move to the next pair in the list, that is t←t+2.

#### 4.10.2 Compliance with breeding recommendations

There are many reasons that breeders can be unable to comply with breeding recommendations made purely on quantitative features. These include practicalities of the geographic locations of the animals, properties of the captive individuals such as mate incompatibility, and external knowledge of suitability not encoded in the breeding model. To simulate these effects we introduce a compliance rate *c* when performing the ranking in Section 4.10.1. In stage 4, when performing pairing, with probability *c* we follow the recommendation, and otherwise (with probability 1−*c*) we instead choose an unpaired individual (lower ranked) uniformly at random, and skip the current individual in the ranking. We examined *c* = 0.6 and *c* = 0.8.

Because fit individuals fail to mate with probability *c*, this is a strong penalty. In a real breeding program, non‐compliance with a *specific mating pair* might be common (and may be 60% or lower), but the breeders are likely to find alternative good solutions that still involve that individual mating with another fit individual. Therefore, we believe that this model is likely to be representative for larger *c*.

#### 4.10.3 Threshold score breeding rule

A simpler process is to rank all individuals according to the selected score in the current generation, select all individuals above some threshold and then perform random mating with the selected individuals. We use a threshold value as the 50% quantile as a sensitivity analysis to the details of the breeding procedure, and the results are qualitatively the same; see Figure S3.

### 4.11 Scottish wildcat data

The sequencing data from (Jamieson et al., 2023) were used by (Howard‐McCombe et al., 2023) where the data were analysed with MOSAIC (Salter‐Townshend & Myers, 2018) to infer the ‘local ancestry’ that is the population source (wildcat or domestic cat, as determined by outgroup individuals and historical wildcat data) for every SNP for each individual. Briefly, the pipeline used Bowtie2 (McKenna et al., 2010) for alignment to the reference (checking that the alignment rates are not affected by wildcat/domestic/hybrid status), and variants were called and filtered with GATK (Langmead & Salzberg, 2012). Relatives were removed from the reference with PLINK2 http://www.cog‐genomics.org/plink/2.0/(kinship > 0.125). Phasing was performed with Beagle v5.2 (Browning & Browning, 2007) and variants were then thinned at random to one SNP per 2 kb to reduce the computational burden. We selected chromosome E2 from this dataset, resulting in 17,720 SNPs for 36 Scottish wildcat and hybrid individuals.

The expected number of offspring per pair per generation is calculated from data matched to the stud book of the breeding program (Barclay, 2019) as follows. Firstly, the observed offspring distribution per brood in the stud book is (0.23, 0.36, 0.30, 0.10, 0.01) for (1, 2, 3, 4, 5) kittens respectively, with a mean of 2.3. 30% of individuals survive to first year and average mortality is then 10% per year thereafter, with 50% of adult females bred per year, leading to a breeding probability for ages a=1,…,7 of $0.5\;\left(1-0.3\right){\left(1-0.1\right)}^{a-1}$ with an expected number of breeding attempts of 1.83. Multiplying by the mean brood size leads to an expected number of offspring of 4.2, which we assume to be Poisson distributed.

## 5 AUTHOR CONTRIBUTIONS

DJL designed and performed the research, analysed data and wrote the paper. JH analysed data and helped write the paper. MB and HS designed the research and helped write the paper.

## CONFLICT OF INTEREST STATEMENT

The authors declare no conflict of interest.

## CODE AVAILABILITY

All code used in this project is available at https://github.com/danjlawson/localsancestrybreeding.

## Supporting information


Figure S1

Figure S2

Figure S3

## Data Availability

Data sharing is not applicable to this article as no new data were created or analysed in this study.
