# Supplementary figures and images for: How admixed captive breeding populations could be rescued using local ancestry information
Source: Mol Ecol. 2024 Apr 18;34(19):e17349. doi: 10.1111/mec.17349 (PMC12456114; doi:10.1111/mec.17349)

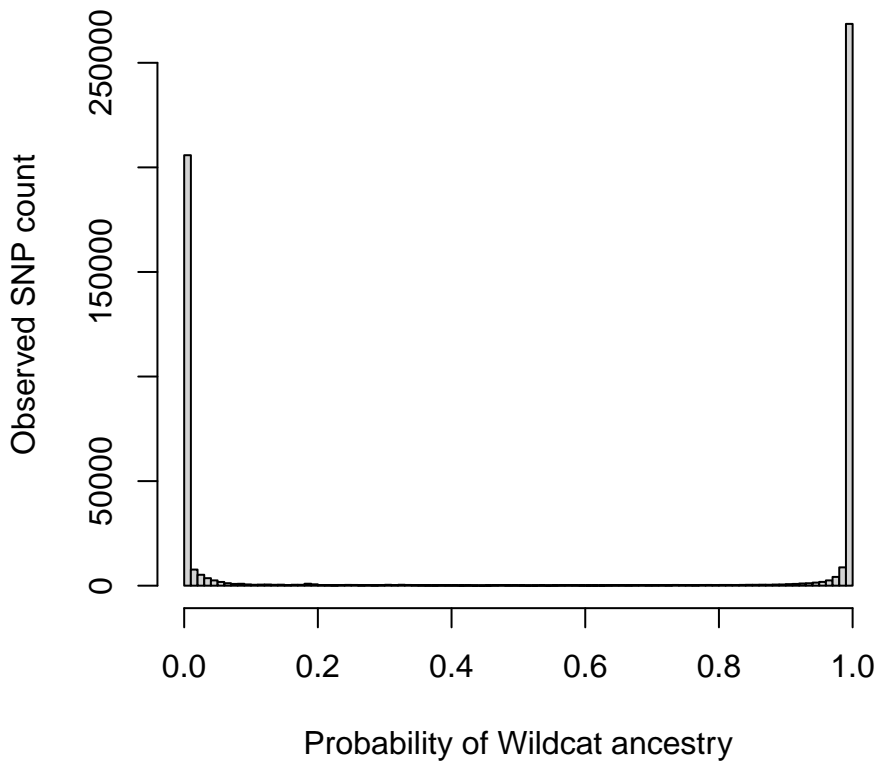

Supplement: Supplementary file 1 — Figure S1 [file MEC-34-e17349-s002.pdf]

### a) Selecting Population Kinship (PK)

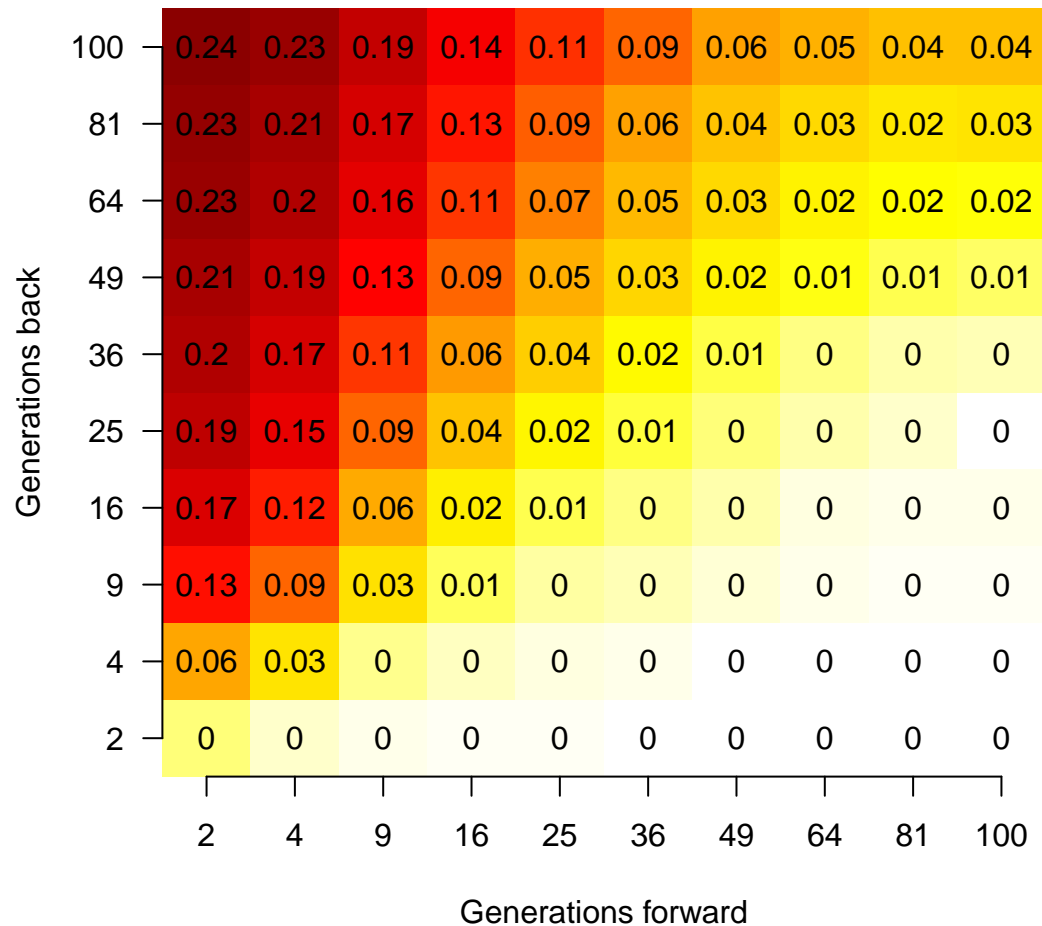

### b) Selecting Weighted Population Het. (WPH)

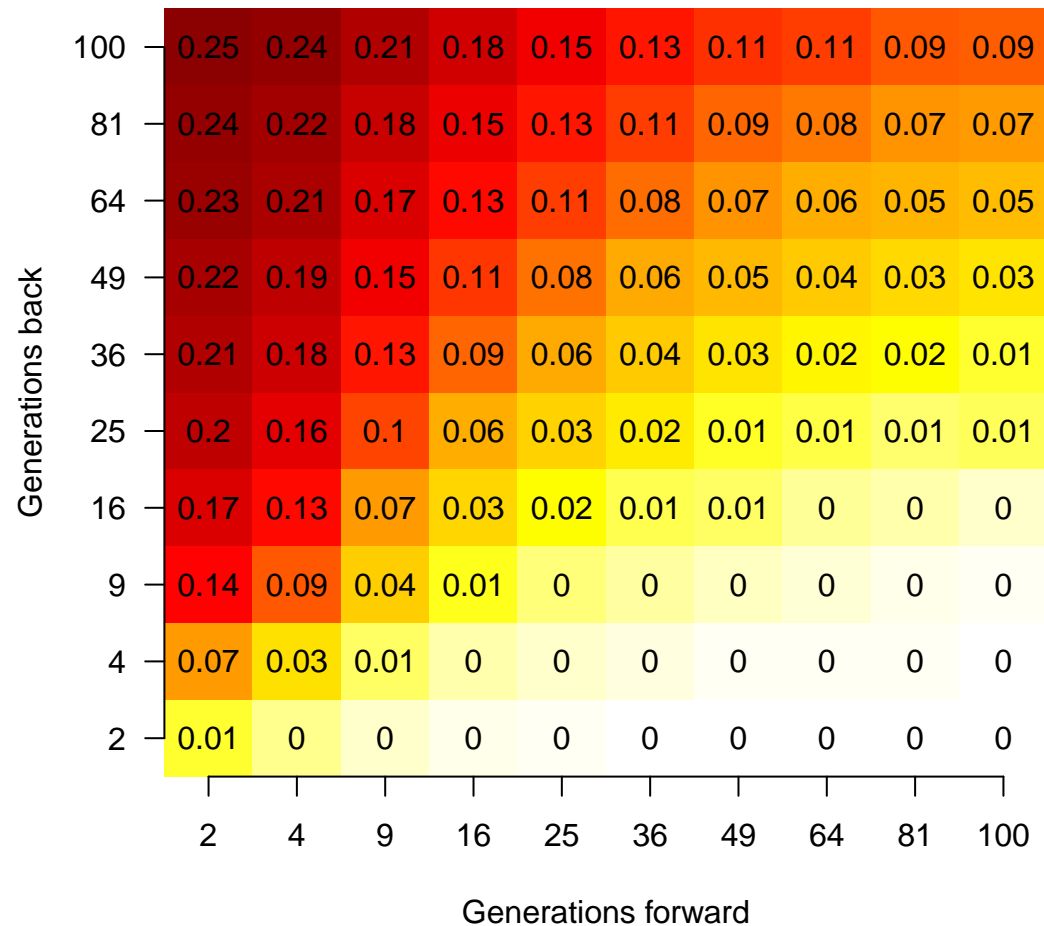

Supplement: Supplementary file 2 — Figure S2 [file MEC-34-e17349-s001.pdf]
