# Supplementary material for: How admixed captive breeding populations could be rescued using local ancestry information
Source: Mol Ecol. 2024 Apr 18;34(19):e17349. doi: 10.1111/mec.17349 (PMC12456114; doi:10.1111/mec.17349)
Supplement: Supplementary file 3 — Figure S3 [file MEC-34-e17349-s003.pdf]

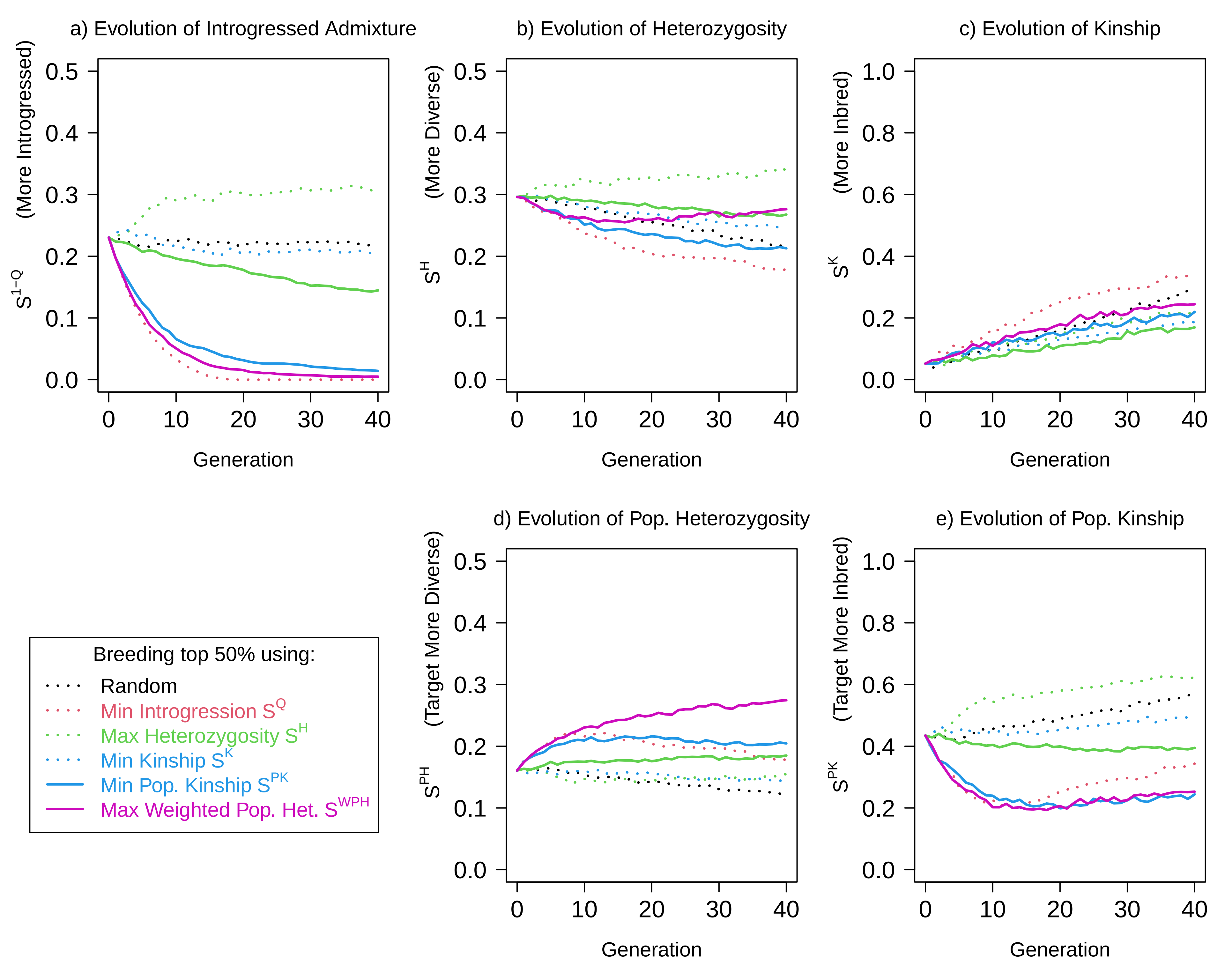
a) Evolution of Introgressed Admixture
(More Introgressed)
S^{1-Q}
b) Evolution of Heterozygosity
(More Diverse)
S^H
c) Evolution of Kinship
(More Inbred)
S^K
d) Evolution of Pop. Heterozygosity
(Target More Diverse)
S^{PH}
e) Evolution of Pop. Kinship
(Target More Inbred)
S^{PK}
Generation
Breeding top 50% using:
Random
Min Introgression S^Q
Max Heterozygosity S^H
Min Kinship S^K
Min Pop. Kinship S^{PK}
Max Weighted Pop. Het. S^{WPH}
